# Changes in the Epidemiology of Rubella: The Influence of Vaccine-Introducing Methods and COVID-19

**DOI:** 10.3390/vaccines11081358

**Published:** 2023-08-12

**Authors:** Naruhito Otani, Masayuki Shima, Takashi Ueda, Kazuhiko Nakajima, Yoshio Takesue, Takuma Yamamoto, Toshiomi Okuno

**Affiliations:** 1 Department of Public Health, Hyogo Medical University, Nishinomiya 663-8501, Hyogo, Japan; 2 Department of Infection Control and Prevention, Hyogo Medical University, Nishinomiya 663-8501, Hyogo, Japan; 3 Department of Legal Medicine, Hyogo Medical University, Nishinomiya 663-8501, Hyogo, Japan; 4 Department of Microbiology, Hyogo Medical University, Nishinomiya 663-8501, Hyogo, Japan

**Keywords:** congenital rubella syndrome, epidemiology, rubella, vaccine

## Abstract

Rubella is an infectious disease caused by the rubella virus. Congenital rubella syndrome is a risk for all newborns if pregnant women are infected with rubella, raising an important public health issue. Rubella is a vaccine-preventable disease, and routine immunization has been conducted in Japan. The timing of the vaccine approval did not differ from that in the United States. In 2004, endemic rubella was eliminated in the United States. However, recent rubella outbreaks have occurred in Japan. This may be related to differences in the introduction of routine rubella immunization. In Japan, routine rubella immunization was initially introduced only for junior high school girls, and the rate of susceptibility is high among males who have not received rubella vaccination, causing an outbreak. Therefore, in Japan, measures have been taken to decrease the number of susceptible males in the vaccination-free generation. The coronavirus pandemic has also affected the epidemiology of rubella as well as other infectious diseases.

## 1. Introduction

Rubella is an acute disease caused by the rubella virus and characterized by fever, exanthema, and lymph node swelling. Clinically, this disease is mild; however, primary infection with the rubella virus in the initial phase of pregnancy results in congenital rubella syndrome (CRS), which is important from the viewpoint of public health. The rubella virus is the most common infectious cause of birth defects. Approximately 100,000 infants worldwide may be born with CRS each year [1]. Rubella outbreaks may increase the morbidity rate in cases of CRS.

The rubella vaccine was approved in the United States in 1969 [2]. It was approved for use in Japan in 1975. Rubella vaccination aims to eliminate both rubella and CRS. There are two approaches for implementing rubella vaccines [3]. The first approach is to perform preventive vaccination in adolescent girls, females of childbearing age, or both for personal prevention. This method focuses on reducing the incidence of CRS. The second approach is more comprehensive and blocks rubella virus transmission and eliminates rubella and CRS by combining the introduction of the rubella vaccine into childhood routine immunization programs with preventive vaccination for highly susceptible older age groups [3]. The first approach was adopted in Japan and the United Kingdom, while the second approach was adopted in the United States; disease management strategies differed among countries [4]. The rubella vaccine was introduced using different approaches in the United States and Japan. In the United States, the elimination of endemic rubella was declared in 2004, while outbreaks of rubella have recently occurred in Japan. In this review, epidemiological data were organized using data published by the National Institute of Infectious Diseases (NIID) in Japan and the Centers for Disease Control and Prevention (CDC) in the United States as available on their respective websites. We present the changes in the epidemiology of rubella and CRS after the introduction of rubella vaccines in Japan and the United States, as well as discuss the concept of rubella immunity. Finally, we discuss the influence of coronavirus disease 2019 (COVID-19) on other infectious diseases.

## 2. Japan

### 2.1. Changes in the Rubella Vaccination System and Target Population

In June 1976, rubella was covered by routine immunization. In Japan, a method for vaccinating adolescent girls has been adopted, focusing on the prevention of infection during pregnancy. In August 1977, routine rubella immunization was initiated for junior high school girls [5]. In April 1989, the measles, mumps, and rubella (MMR) vaccination was administered to boys and girls aged 12–90 months instead of the measles vaccine alone. However, in April 1993, aseptic meningitis occurred frequently, and the MMR vaccination was discontinued. In April 1995, rubella vaccination was initiated in boys and girls aged 12–90 months. Routine immunization was performed in junior high school boys and girls whose generations did not correspond to the routine immunization age until 2003 [5]. Furthermore, in 1995, mandatory mass vaccination was changed to an individual opt-in strategy with the enforcement of an amended preventive vaccination law [6]. In 2006, the one-dose rubella vaccination was changed to a two-dose measles-rubella (MR) vaccination (first dose: 1 year after birth, second dose: 1 year before entrance into elementary school). Between 2008 and 2012, the opportunity for a second-dose MR vaccination was introduced at 12–13 and 17–18 years of age [7]. In December 2018, preventive rubella vaccination was administered after confirming susceptibility in males born between April 2, 1962, and April 1, 1979, with no opportunity for routine rubella immunization apart from routine immunization for children [8]. A summary of this process is given in Table 1, including a comparison with the United States vaccination programs.

### 2.2. Vaccination Rates for Rubella in Japan

The vaccination rate among junior high school girls at the start of routine immunization in August 1977 was 27.3%. This percentage may have ranged from 60 to 70% between 1978 and 1989 [9]. However, during this period, junior high school boys were rarely vaccinated; therefore, the overall vaccination rates for boys and girls will have been remarkably low.

In 1995, routine immunization for young children aged 12–90 months and junior high school students who had not received routine immunization began; however, vaccination rates in young children and junior high school students in 1997 were 60 and 46%, respectively [6]. In 2001, the vaccination rate among young children was 97%, whereas that among junior high school students was 39%. This rate decreased annually among junior high school students until 2001 [5].

In 2006, a two-dose MR vaccination program was initiated. The first and second vaccination rates in 2010 were 96 and 92%, respectively [10]. The first and second vaccination rates in 2016 were 97.2 and 93.1%, respectively [11]. Additionally, the rates increased in 2018 and were 98.5 and 94.6%, respectively [8]. However, the first and second vaccination rates were lower than the target value (≥95%) for eliminating rubella following COVID-19; the respective rates were 93.5 and 93.8% in 2021 [12].

### 2.3. Epidemiology of Rubella

In 1981, the National Epidemiological Surveillance of Infectious Diseases (NESID) was initiated [13]. However, this did not have any legal basis. The Act on the Prevention of Infectious Diseases and Medical Care for Patients with Infectious Diseases (Infectious Diseases Control Law) was enacted in April 1999. Based on this law, the NESID program was defined as having one of its main objectives [13]. According to the number of reports on rubella published by sentinel clinics in accordance with the NESID program, major epidemics repeatedly occurred in 1982, when the NESID program was initiated, in 1987–1988, and in 1992–1993, that is, every five years [6,14]. However, after 1994, there was no major epidemic for a while [14]. In 2004, an epidemic affecting an estimated 39,000 people occurred [7]. Subsequently, there was no increase over the next 7 years.

In 2008, the Infectious Diseases Control Law was amended, and sentinel surveillance was changed to notifiable disease surveillance [7]. In subsequent outbreaks, the number of patients with rubella increased to 378 in 2011 and further increased to 2386 in 2012 and 14,344 in 2013 [15]. The prevalence of rubella in 2013 showed that many susceptible adult males remain in Japan [15]. Additionally, epidemics involving metropolitan areas occurred in 2018 (*n* = 2941) and 2019 (*n* = 2306). Adults accounted for approximately 95% of all reported cases, and males accounted for approximately 80% [8]. The number of patients decreased to 101 by 2020. It was 12 in 2021 and 15 in 2022 [12,16]. This was possibly related to infection control measures, such as wearing masks, during the COVID-19 pandemic.

### 2.4. Epidemiology of CRS

According to a survey conducted by the Ministry of Health and Welfare, the number of reports on CRS is small, and a Japanese strain of the rubella virus was considered to have lower teratogenicity [17]. However, a subsequent survey indicated that 83 and 232 people, respectively, developed CRS after rubella outbreaks in 1965–1969 and 1975–1977 [18,19]. According to another survey, the incidences were 0.2–8.1/100,000 live births in an epidemic year and 0.1–0.7/100,000 live births in a non-epidemic year [20]. These results show that the incidence of CRS in Japan is similar to that in the United States. According to a questionnaire survey, 639 patients were reported between 1963 and 1992 and 301 between 1978 and 1993 [6].

After the enforcement of the Infectious Diseases Control Law in 1999, no patients with CRS were reported. One patient was reported annually between 2000 and 2003 [5]. During the epidemic period (2003–2004), 10 patients with CRS were reported [7]. Three cases of CRS were reported between 2008 and 2011 [21]. During a rubella outbreak from 2011 to 2013, 45 patients with CRS were reported between 2012 and 2014 [15]. There were no reports from 2015 to 2018. However, with an additional rubella outbreak, four patients with CRS were reported in 2019 and one in 2020 [8].

### 2.5. Rubella-Susceptible Persons

The herd immunity threshold for rubella is reportedly 83–85% [22,23]. According to the results of an antibody test involving 3164 persons (1783 males and 1381 females) in Japan in 2020, the antibody prevalence rate with a rubella HI antibody titer of 1:8 or higher was approximately ≥90% in both males and females aged 2–34 years. Among females aged 35–59 years, it was ≥90% in all age groups. However, in males aged 40–59 years, it was approximately 80%, which is below the threshold for achieving herd immunity [24]. In Japan, the number of immune individuals necessary to acquire herd immunity has not been reached in men aged 40–59 years. In fact, the number of infected males in this age group was high during the outbreaks in 2013, 2018, and 2019 [15]. This was possibly because routine rubella immunization was performed only on junior high school girls in this generation. Since December 2018, measures have been taken to offer preventive vaccination to males who had previously not received routine rubella vaccination and had low antibody titers against rubella in this age group [8].

## 3. United States

### 3.1. Changes in the Vaccination System in the United States

In the United States, live-attenuated rubella vaccines were first approved in 1969 (Table 1) [25]. The new rubella vaccination program involved single-dose vaccination in 1 year-old to adolescent children [10]. In 1978, the Advisory Committee on Immunization Practices recommended that rubella vaccine targets should include highly susceptible postpubertal females, adolescents, college students, persons in military service, and persons working at specific workplaces, such as hospitals, based on epidemiological changes in rubella [26]. Each state’s immunization plans were established, and preschool vaccination became obligatory [27]. In 1990, a new schedule for a two-dose vaccination was recommended [27].

### 3.2. Vaccination Rate

The vaccination rates before 1977 were approximately 60% in children aged 1–4 years, 71% in those aged 5–9 years, and 64% in those aged 10–14 years [4]. The vaccination rate of children entering school (i.e., kindergarten or first grade) in 50 states and the District of Columbia from 1981 to 1982 was 96% [28]. Such high vaccination rates in these areas may be associated with the enforcement of school immunization laws [26]. A total of 94.8% of kindergarteners in 2009–2010 and 90.5% of adolescents in 2010 had evidence of a two-dose vaccination [29]. The two-dose MMR vaccination rate from 2021 to 2022 was 93.0% [30].

### 3.3. Epidemiology of Rubella

During the rubella epidemic from 1962 to 1965, approximately 12,500,000 people were infected with rubella, and approximately 2000 people developed encephalitis. Reportedly, 11,250 fetuses and 2100 neonates died [26,31]. Rubella and CRS were reported nationally in 1966. The CDC established the National Congenital Rubella Syndrome Registry (NCRSR) in 1969 [4]. Since the start of the rubella vaccination program in 1969, there has been no epidemic similar to the one that occurred from 1962 to 1965 [4]. In 1969, when the vaccination started, 57,686 patients were reported, but the number of patients decreased by 78% to 12,491 in 1976 [26]. Most patients with rubella were <15 years of age before the start of vaccination. However, after the start of vaccination in 1969, the number of patients with rubella decreased until 1977. The rate of decrease was the greatest in individuals aged <15 years, whereas there was no decrease in those aged ≥15 years; they accounted for 76.1% of all patients [25]. In the 1980s, the number of patients with rubella decreased steadily, and its incidence in all age groups continued to decrease. In 1983, 970 patients were diagnosed with rubella [26]. There was a decrease from 0.45/100,000 people in 1990 to 0.1/100,000 people in 1999 [32]. Furthermore, most patients with rubella before 1995 were non-Hispanic, whereas those diagnosed between 1995 and 1999 were Hispanic [32]. The number of patients with rubella in 2001, 2002, 2003, and 2004 was 23, 18, 7, and 9, respectively. In 2004, the elimination of endemic rubella was declared in the United States [26]. A total of 54 patients with rubella were reported between 2004 and 2009; however, the disease was associated with imports in all patients [29].

### 3.4. Epidemiology of CRS

During the rubella epidemic from 1962 until 1965, approximately 20,000 infants with CRS were born [26]. In 1969, a vaccination program was started. The number of infants with CRS decreased from 68 in 1970 to 23 in 1976. In 1983, it was four [26]. According to the data from the NCRSR, the incidence of CRS decreased from 2.7 cases per 100,000 births to 1.0 case per 100,000 births between 1969 and 1979 [4]. Of the 24 infants with CRS born between 1997 and 1999, 20 were born to Hispanic mothers. Of these 23 infants, 21 were born to mothers who were born abroad [32]. Furthermore, four infants with CRS were reported between 2001 and 2004. In three of these cases, the mother was born in a country other than the United States [26]. Four infants with CRS were reported between 2005 and 2009. In three of these cases, CRS was acquired internationally [29].

## 4. Evidence on Rubella Immunity

According to the CDC recommendations [33,34], acceptable presumptive evidence of immunity against rubella includes at least one of the following: (i) written documentation of vaccination with one dose of live rubella virus-containing vaccine administered on or after the first birthday, (ii) laboratory evidence of immunity, (iii) laboratory confirmation of rubella disease, or (iv) birth before 1957 (except women who could become pregnant). Generally, serological examination before and after vaccination is not recommended; however, it is described that additional MMR vaccination (a maximum of three doses) should be offered when rubella-specific IgG-positive reactions are not clear in women of childbearing age who have received rubella vaccination once or twice and that additional serological examination is not necessary [33]. When conducting a serological examination, the cut-off value for positive/negative reactions for detecting rubella antibodies is ≥10 IU/mL [2,35]. In Japan, the two-dose vaccination started in 2006, and the interval from that point was short; therefore, serological examination-based evaluations are frequently performed.

More than 95% of individuals develop immunity after single-dose rubella vaccination, and long-term persistence of protection is achieved. However, individuals are vaccinated with the rubella vaccine in combination with other vaccines, including that for measles, and the two-dose vaccination is often conducted for vaccine program-related reasons [1].

The criteria for acceptable evidence of active rubella immunity are used as guidelines for the evaluation of vaccination and administration in clinical practice and public health settings. Active immunity is used as a tool to provide presumptive evidence but not absolute evidence of immunity [33]. In other words, individuals meeting the criteria may have acquired immunity but may have been reinfected with rubella [33]. There are two methods of acquiring immunity: natural infection-medicated methods and vaccination-mediated methods. One study suggested that individuals acquiring immunity through vaccination are more readily reinfected than those acquiring immunity through natural infection [36].

## 5. Diagnosis

### 5.1. Rubella

Diagnostic methods include serological diagnosis, RNA detection, and virus isolation.

Rubella virus-specific IgM (rubella IgM) or IgG (rubella IgG) were used for serological diagnosis. Rubella IgM is detected after a primary infection or vaccination. Rubella IgG can be detected 4 days after the appearance of rubella-related exanthema, and it subsequently reaches a peak in 1 to 2 weeks. Rubella IgM may persist for 3 months, while rubella IgG may persist over the lifetime [34]. Based on these antibody properties, rubella can be diagnosed based on the detection of IgM antibodies in the acute phase, pair-serum-related IgG antibody seroconversion in the acute and recovery phases, or a ≥4-fold increase in IgG antibody levels [34,37]. Pharyngeal swabs, blood, and urine were extracted to detect RNA using polymerase chain reaction (PCR) [34,38]. The positivity rate is increased by collecting samples as early as possible after onset, 0–3 days after the appearance of exanthema if possible, and within 7 days after onset at the latest [11,34]. A combination of PCR and IgM antibody tests 0 to 3 days after the appearance of exanthema, during which specific IgM detection may result in false-negative reactions, may facilitate a more accurate examination/diagnosis. In Japan, it was necessary to measure the serum antibody titer and submit samples for viral genome testing in all rubella-suspected patients in 2018, and the laboratory diagnosis rate increased from 63 to 78% between 2013 and 2017 to approximately 95% from 2018 to 2019 [8].

### 5.2. CRS

CRS can be diagnosed if rubella IgM antibodies are detected within 6 months after birth or if there is an increase in the rubella IgG antibody titer 7–11 months after birth, that is, before rubella vaccination [34,37].

However, the specificity of rubella IgM testing is not 100%, and false-positive reactions have been detected in some cases. The measurement of IgM antibodies during pregnancy should be limited to rubella-suspected patients [34]. Rubella IgG avidity is sometimes measured to distinguish between recent and historical infections. Antibody avidity serially increases, and low-avidity rubella IgG suggests a recent infection, whereas high-avidity rubella IgG suggests a past infection [34,37]. Therefore, when high-avidity rubella IgG is detected despite a rubella IgM-positive reaction, the rubella IgM-positive reaction may be evaluated as a false positive.

## 6. Status of Rubella Worldwide

### 6.1. Vaccination Rate

The number of countries where the rubella vaccine was introduced into routine immunization programs was 99 in 2000, which increased to 173 in 2019 [1]. The bureaus of the World Health Organization (WHO) are located in six areas of the world (Africa, America, Southeast Asia, Europe, the Eastern Mediterranean, and the Western Pacific). According to the Global Vaccine Action Plan 2020, rubella was eliminated only in America as of 2019 [39]. Furthermore, rubella transmission was eliminated in 93 out of 194 principal countries [40]. With respect to WHO regions, the vaccination rates in 2019 in the African, American, Southeast Asia, European, the Eastern Mediterranean, and the Western Pacific regions were 33, 88, 93, 96, 45, and 94%, respectively. The overall vaccination rate is 71%. This figure has increased from 35% in 2010 [39].

### 6.2. Epidemiology

The number of rubella-infected people per 1,000,000 people worldwide decreased from fifteen in 2010 to seven in 2019 [39]. In 2020, rubella surveillance was conducted in 194 countries, of which 193 had standardized, quality-controlled laboratory testing through the WHO Global Measles and Rubella Laboratory Network. A total of 175 countries reported the number of rubella cases in 2012, and this number increased to 179 in 2019. However, this number decreased to 135 in 2020 during the COVID-19 pandemic [40]. The number of reported patients was 94,277 in 2012, but it decreased to 49,136 in 2019 and 10,194 in 2020 [40]. The decrease in the number of patients reported in 2020 may have been associated with a reduction in surveillance; however, strategies against COVID-19, such as social distancing and wearing masks, may have been useful in preventing the spread of the rubella virus [41].

## 7. COVID-19 Related Changes in the Epidemiology of Other Infectious Diseases

With the onset of the COVID-19 pandemic in 2020, various infection control strategies have been implemented. Under the influence of these strategies, there have been changes in the epidemic conditions of infectious diseases other than COVID-19. In the United States, the state or timing of the respiratory syncytial virus (RSV) epidemic has changed. No epidemics occurred during the 2020–2021 season. There was an epidemic during the 2021–2022 season, but the timing of the epidemic differed from that of the pre-pandemic period [42]. An out-of-season RSV epidemic may be related to a reduction in herd immunity associated with long-term low-level exposure to RSV, which is termed “immunity debt [43,44]”. Non-pharmaceutical interventions (NPI) during the COVID-19 pandemic may induce immunity debt, exhibiting negative effects during pandemic convergence [44]. Similarly, no RSV epidemics occurred during the 2020–2021 season in Japan. However, an epidemic was observed in the 2021–2022 season, with a marked increase in the number of cases compared with that during the pre-pandemic periods. [45,46]. In infectious diseases, such as RSV, which affect most humans during childhood and confer immunity upon infection, the impact of NPI-related immunity debt may be pronounced. There were some infectious diseases other than RSV infection whose epidemics decreased during the pandemic period. These diseases included mumps. Mumps epidemics occur every 4–5 years in Japan, but there have been no epidemics since the COVID-19 pandemic [47]. In Japan, the routine immunization program does not involve the mumps vaccine, and the vaccination rate is low. Currently, the number of mump-susceptible individuals may increase, and an outbreak may occur after NPI withdrawal. Concerning influenza, there were no epidemics during the 2020–2021 or 2021–2022 seasons in the United States. However, during the 2022–2023 season, an epidemic at the pre-COVID-19 pandemic level occurred [48]. In Japan, there were no epidemics during the 2020–2021 or 2021–2022 seasons. However, there was a specific epidemic during the 2022–2023 season, although it did not reach pre-pandemic levels [49]. In the future, the effects of pathogen interference may have an impact on epidemiology [50]. In Japan, NPI was relatively relaxed in the spring of 2023; therefore, an influenza outbreak at the pre-COVID-19 pandemic level may occur in the following season.

## 8. Conclusions

Rubella vaccines have been developed and approved for use in several countries. Subsequently, various rubella vaccination programs have been initiated. Immunization of adolescent girls or females of childbearing age alone does not influence the epidemiology of rubella. As rubella infection occurs before the age of immunization in most cases, it would be necessary to vaccinate all susceptible females to eliminate CRS [51]. Furthermore, virus circulation is reduced, and susceptibility remains until adolescence or adulthood in some individuals who did not undergo vaccination and were not infected during childhood [1]. When childhood immunization was performed in both sexes, the mean age of patients with rubella increased, but its incidence in all age groups decreased when the vaccination rate was high. However, when the vaccination rate is low, the circulating virus cannot be disrupted, thereby increasing the susceptibility of women of reproductive age [1]. Therefore, it is important to achieve and maintain high vaccination rates.

In the United States, the rubella vaccine was introduced into routine immunization programs during childhood. The elimination of endemic rubella was declared in 2004. Cases of CRS were observed in patients who were associated with immigrants from countries where routine immunization programs were not consistently implemented. In contrast, in Japan, the incidence of rubella has decreased, but outbreaks have also recently occurred. In Japan, a program to vaccinate adolescent females was initially selected with a focus on the prevention of infection in pregnant women. However, the rubella vaccine was only introduced into the routine immunization program during childhood after the start of this program. Consequently, outbreaks involving non-vaccinated males are still observed in Japan. During the COVID-19 pandemic, NPI decreased the number of patients with rubella, but this did not reflect a decrease in the number of susceptible persons; an epidemic may occur in the future.

In Japan, there has been a reduction in the rubella vaccination rate during the COVID-19 pandemic, raising concern. These problems are also common among other vaccine-preventable diseases (VPDs), whose outbreaks have decreased during the COVID-19 pandemic. Therefore, promoting vaccination against VPDs is necessary.

## Figures and Tables

**Table 1:** Timeline of evolving rubella vaccine strategies.

Year	Changes in Immunization Programs in the United States and Japan	
	The United States	Japan
1969	Approval of the rubella vaccine	
	Start of single-dose vaccination for 1 year-old to pubertal children.	
1975		Approval of the rubella vaccine
1977		Start of vaccination for junior high school girls
1989		Start of single-dose vaccination for boys and girls aged 12 to 90 months.
1990	Start of a two-dose vaccination at 12 to 15 months and 4 to 6 years of age.	
1995		Single-dose vaccination for boys and girls aged 12 to 90 months
		Single-dose vaccination for junior high school boys and girls (during a limited period of April 1995 to September 2003).
2004	Declaration on the elimination of endemic rubella.	
2006		Start of a two-dose vaccination for children aged 12 to <24 months and aged 5 to <7 years
2008		Second vaccination for children aged 12 to 13 years and aged 17 to 18 years (during a limited period of 2008 to 2012).
2015	Declaration on the achievement of rubella and CRS elimination (Region of the Americas).	
2019		Single-dose vaccination for susceptible males who were born between 2 April 1962, and 1 April 1979 (continued).

## Data Availability

Not applicable.
